# Molecular analysis of pyrazinamide resistance in *Mycobacterium tuberculosis* in Vietnam highlights the high rate of pyrazinamide resistance-associated mutations in clinical isolates

**DOI:** 10.1038/emi.2017.73

**Published:** 2017-10-11

**Authors:** Nguyen Quang Huy, Contamin Lucie, Tran Thi Thanh Hoa, Nguyen Van Hung, Nguyen Thi Ngoc Lan, Nguyen Thai Son, Nguyen Viet Nhung, Dang Duc Anh, Bañuls Anne-Laure, Nguyen Thi Van Anh

**Affiliations:** 1 UMR MIVEGEC (5290 CNRS-224 IRD-Université de Montpellier), Institute of Research for Development, 34394 Cedex 5 Montpellier, France; 2 Department of Bacteriology, National Institute of Hygiene and Epidemiology (NIHE), Hanoi 1000, Vietnam; 3 Department of Pharmacological, Medical and Agronomical Biotechnology, University of Science and Technology of Hanoi, Hanoi 1000, Vietnam; 4 LMI Drug Resistance in South East Asia, Hanoi 1000, Vietnam; 5 Department of Microbiology, National Lung Hospital, Hanoi 1000, Vietnam; 6 Department of Microbiology, Pham Ngoc Thach Hospital, Ho Chi Minh 7000, Vietnam; 7 Department of Microbiology, Military Medical University, Hanoi 1000, Vietnam; 8 Vietnam National Tuberculosis Control Program, Hanoi 1000, Vietnam

**Keywords:** MIRU-VNTR, multidrug resistance, *pnc*A mutation, pyrazinamide resistance, *Mycobacterium tuberculosis* family, sequencing, spoligotyping

## Abstract

Pyrazinamide (PZA) is a key antibiotic in current anti-tuberculosis regimens. Although the WHO has stressed the urgent need to obtain data on PZA resistance, in high tuberculosis burden countries, little is known about the level of PZA resistance, the genetic basis of such resistance or its link with *Mycobacterium tuberculosis* families. In this context, this study assessed PZA resistance through the molecular analysis of 260 Vietnamese *M. tuberculosis* isolates. First-line drug susceptibility testing, *pnc*A gene sequencing, spoligotyping and mycobacterial interspersed repetitive units-variable number of tandem repeats (MIRU-VNTR) typing were performed. Overall, the *pnc*A mutation frequency was 38.1% (99 out of 260 isolates) but was higher than 72% (89 out of 123 isolates) in multidrug and quadruple-drug resistant isolates. Many different *pnc*A mutations (71 types) were detected, of which 55 have been previously described and 50 were linked to PZA resistance. Among the 16 novel mutations, 14 are likely to be linked to PZA resistance because of their mutation types or codon positions. Genotype analysis revealed that PZA resistance can emerge in any *M. tuberculosis* cluster or family, although the mutation frequency was the highest in Beijing family isolates (47.7%, 62 out of 130 isolates). These data highlight the high rate of PZA resistance-associated mutations in *M. tuberculosis* clinical isolates in Vietnam and bring into question the use of PZA for current and future treatment regimens of multidrug-resistant tuberculosis without PZA resistance testing.

## INTRODUCTION

Pyrazinamide (PZA) is a crucial first-line drug (FLD) for tuberculosis (TB) treatment because it shortens the treatment duration in patients with susceptible, multidrug-resistant (MDR, isolates resistant to at least isoniazid and rifampicin) or extensively drug-resistant (XDR, MDR isolates resistant to any fluoroquinolone and at least one second-line injectable drug) TB and reduces TB relapse rates.^1, 2^ The powerful sterilization activity of PZA allows it to kill persistent tubercle bacilli in macrophages or in the acidic environment of lesions that are not eradicated by other anti-TB drugs.^3^ PZA is the only FLD that is most likely to be maintained in all new regimens for shortening the treatment course of all forms of TB.^4, 5^ Altogether with second-line anti-TB drugs, the use of PZA in the treatment of MDR TB cases has significantly improved the success of anti-TB therapy.^6, 7^ Culture-based PZA susceptibility testing is difficult to perform and produces unreliable results due to the need for an acidic pH medium that inhibits bacterial growth and for large inoculum volumes that might reduce PZA activity.^8, 9^ The automated Bactec MGIT 960 liquid culture system (Sparks, MD) is the only method recommended by the World Health Organization (WHO) for phenotypic-PZA susceptibility testing.^5^ Nevertheless, this method is difficult to perform, and still produces a high rate of false-positive resistance results.^9, 10^

Therefore, PZA susceptibility testing is not routinely performed and little is known about PZA resistance in *M. tuberculosis* populations, especially in high TB burden countries.^4^ Nevertheless, a recent study performed in five high TB and MDR TB burden countries reported PZA resistance rates that ranged from 3.0% to 42.1%.^11^

Although several mechanisms have been described, PZA resistance is mostly caused by mutations in the *pnc*A gene and its promoter that lead to a reduction or loss of pyrazinamidase (PZase) activity.^12, 13, 14, 15, 16^ Many different *pnc*A mutations have been described and are scattered throughout the *pnc*A gene with no one mutation predominating.^12, 17, 18^ Nevertheless, the three regions (codons 3–17, 61–85 and 132–142) that contain the PZase active and metal-binding sites show some degree of mutation clustering.^14, 19^ Mutations in the *pnc*A promoter can also affect PZase activity by disturbing *pnc*A gene translation.^12, 14^ The *pnc*A mutation frequency in PZA-resistant *M. tuberculosis* isolates varies between 46% and 97%.^19, 20, 21, 22, 23, 24^ This variability could be attributed to the unreliable results of PZA susceptibility testing, as mentioned above. Recent reviews reported that, globally, over 80% of PZA-resistant isolates harbor mutations in the *pnc*A gene and promoter, whereas up to 10% have no mutation or carry mutations not associated with phenotypic resistance.^17, 18^ Mutations in *pnc*A have also been detected in 9% of PZA-sensitive isolates.^12, 18^

In Vietnam, a country with a high MDR TB burden, little is known about the PZA resistance level. In addition, the correlation between *pnc*A mutations, PZA resistance and *M. tuberculosis* families has not been yet investigated. In the framework of the Vietnam National TB Control Program, PZA has been used to treat new TB cases and relapses since 1990, and has also been added to regimens for patients with MDR or XDR TB since 2009.^25^ However, no information on PZA resistance could be collected during the last national drug resistance survey.^26^ The WHO has highlighted the urgent need to obtain data on PZA resistance in different settings to limit the risk of using and introducing ineffective TB treatment regimens (http://www.who.int/tb/features_archive/TB_resistance_survey_2016/en/).

Therefore, in this study, we assessed PZA resistance in 260 drug-resistant or sensitive *M. tuberculosis* clinical isolates collected in Vietnam by sequencing the *pnc*A gene and identifying PZA resistance-associated mutations. To this end, we determined the rate of *pnc*A gene and promoter mutations in these isolates and their distribution according to the *M. tuberculosis* families, mycobacterial interspersed repetitive units-variable number of tandem repeats (MIRU-VNTR) genotypes and FLD resistance patterns (isoniazid, rifampicin, streptomycin and ethambutol resistance).

## MATERIALS AND METHODS

### *M. tuberculosis* isolates and drug susceptibility testing

Isolates were selected from the culture collection of the Laboratory of Tuberculosis, National Institute of Hygiene and Epidemiology, Hanoi, Vietnam. *M. tuberculosis* samples were collected in three regional TB reference hospitals, the National Lung Hospital (North), Pham Ngoc Thach Hospital (South) and Hue Central Hospital (Central), between 2005 and 2009. As the objective of this study was to evaluate the risk of PZA resistance in sensitive and drug-resistant *M. tuberculosis*, isolates were chosen according to the FLD susceptibility patterns (isoniazid, rifampicin, streptomycin and/or ethambutol resistance) and *M. tuberculosis* families (Table 1). All of the available drug resistance patterns were included. For each FLD resistance pattern (mono to quadruple resistance), we selected isolates according to their *M. tuberculosis* family, determined by spoligotyping. For each family, when the number of isolates with a specific FLD resistance pattern was <ten, all isolates were tested. Conversely, when >ten isolates were available, 10 or more isolates were randomly selected for analysis. In addition, a higher number of MDR isolates were selected to investigate the level of PZA resistance in these particularly problematic clinical isolates. In total, 260 isolates were included in the study. If we consider the percentage of MDR in 2005 (2.7% of new cases and 19% of retreatment cases) and a collection capacity of 1300 strains (see last national survey in 2011),^26^ 335 MDR isolates would have been collected over five years. In our sample, we included 123 MDR isolates that represent 37% of the collection potential to provide a good picture of the MDR *M. tuberculosis* population in Vietnam for that period.

FLD (isoniazid, rifampicin, streptomycin and ethambutol) susceptibility testing was performed using the proportion method, as recommended by the WHO,^27^ at the Vietnamese TB reference laboratories (National Lung Hospital or Pham Ngoc Thach Hospital), or the proportional agar micro-plate assay developed by Nguyen *et al.*^28^ Phenotypic-PZA susceptibility testing was not performed in this study.

### Genomic DNA extraction

A loop full of *M. tuberculosis* colonies grown on Löwenstein–Jensen medium was harvested and suspended in 1 mL of TE buffer (10 mM Tris–HCl, 1 mM EDTA). After incubation at 95 °C for 45 min, bacterial suspensions were centrifuged and DNA-containing supernatants were transferred to new tubes and stored at −20 °C until use.

### Genotyping methods

Spoligotyping was carried out, as previously described.^29^ The results were compared with the SITVITWEB database for *M. tuberculosis* family identification.^30^ The 24-locus MIRU-VNTR technique was used to investigate genotypic diversity and clustering in the selected *M. tuberculosis* samples.^31^ A neighbor-joining based phylogenetic tree was built from DSW distances using MIRU-VNTR *plus* (http://www.miru-vntrplus.org/) (Supplementary Figure S1). Isolates were classified as ‘unknown’ when their spoligotypes could not be identified in the SITVITWEB database and when the 24-MIRU-VNTR typing could not classify them into a family.

### PCR amplification and DNA sequencing

A 709- bp fragment that included 561 bp of the *pnc*A coding sequence, 93 bp of the promoter and 55 bp of the 3′ region was amplified and sequenced using the following primers: F-pncA (5′-CTT GCG GCG AGC GCT CCA-3′) and R-pncA (5′-TCG CGA TCG TCG CGG CGT C-3′) (modified from^19^). Each 25 μL PCR mixture contained 2.5 μL of 10 × reaction buffer, 5 μL of 5 × Q solution, 0.5 μL of 5 mM dNTPs, 0.5 μL of each forward and reverse primer (10 μM), 0.1 μL of 5 U/μL HotStar Taq (QIAGEN, Hilden, Germany), 13 μL of H_2_O and 3 μL of DNA template. The PCR amplification conditions were as follows: 15 min of Taq activation at 95 °C, and then 35 cycles of denaturation at 95 °C, annealing at 63 °C and extension at 72 °C with 1 min for each step, followed by a final extension at 72 °C for 5 min. PCR products were examined in 1.5% agarose gels and sequenced bidirectionally by Eurofins MWG Operon (Germany).

### Sequence and statistical analysis

The *pnc*A sequences were aligned to the *M. tuberculosis* H37Rv reference sequence (GenBank accession number NC_000962.3) to identify mutations using BioEdit version 7.1.10. A two-tailed Fisher’s exact test was used to compare the mutation frequencies according to the drug resistance patterns and the different *M. tuberculosis* families. The odds ratio and 95% confidence interval (95% CI) were calculated to quantify the association of FLD resistance patterns with *pnc*A mutation frequency and the association between the resistance to each FLD (isoniazid, rifampicin, streptomycin and ethambutol) and *pnc*A mutation frequency in our sample. A *P*-value <0.05 was considered statistically significant.

## RESULTS

### FLD susceptibility testing and genotyping

Among the 260 *M. tuberculosis* isolates selected for this study, 55 were susceptible and 205 were resistant to at least one FLD (Table 1). Among the 205 FLD-resistant isolates, 29.8% (*n*=61), 12.3% (*n*=25), 13.7% (*n*=28) and 44.4% (*n*=91) were resistant to one, two, three and four drugs, respectively, and 60% were MDR. Resistant samples could be classified into 11 distinct FLD resistance patterns (Table 1).

The spoligotyping analysis revealed 101 different spoligotypes (Supplementary Figure S1). The Beijing family was the most represented family (50%), followed by the EAI (25.8%) and T (10.8%) families. The ‘Others’ group (13.4%) included unknown spoligotypes (11.9%), LAM (1.2%) and H (0.4%) families.

MIRU-VNTR typing revealed 192 distinct genotypes, of which 163 were unique and 29 were represented by 97 isolates (Supplementary Figure S1). The 24-locus MIRU-VNTR data allowed assigning the eight unknown spoligotypes into an EAI family (named EAI-like). Consequently, the proportion of the EAI family (including EAI and EAI-like genotypes) was 29.2% and that of the ‘Others’ group was 10%.

The combined spoligotyping and 24-locus MIRU-VNTR analyses revealed 209 distinct genotypes, of which 188 were unique and 21 were represented by 72 isolates (average=3.4 isolates/cluster). The largest cluster consisted of 14 isolates with various FLD susceptibility patterns, while the smallest clusters (*n*=14) were composed of only two isolates. The six remaining clusters consisted of 3–8 isolates.

### Analysis of *pnc*A mutations

Among the 260 isolates, 99 (38.1%) carried mutations in the *pnc*A coding region (32.3%) or its promoter (3.9%) or showed the absence of *pnc*A amplification (1.9%) (Table 1 and Supplementary Table S1). In total 71 different mutations were identified, among which 55 had been previously documented, and 16 were new (Supplementary Table S1).^12, 17, 18, 32, 33^

**Figure 1:**
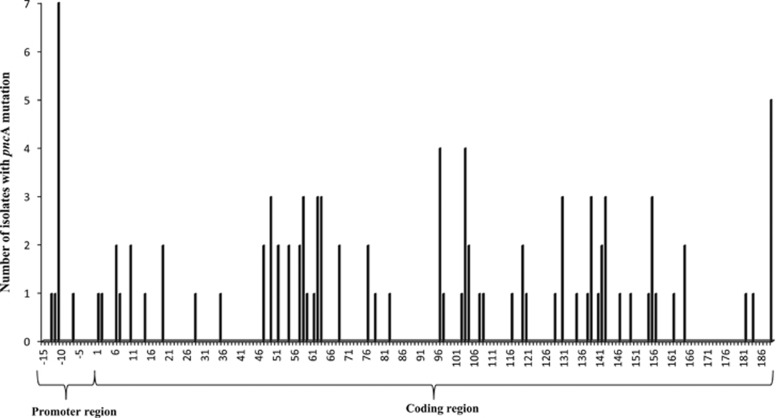
Diversity of *pnc*A mutations in *Mycobacterium tuberculosis* isolates in Vietnam.

Only non-synonymous mutations were present in the *pnc*A coding region and were dispersed throughout the gene. They were found in 50 of the 187 *pnc*A codons (Figure 1). No single mutation was particularly predominant, and the maximum number of isolates with the same mutations was three. The mutation types included single nucleotide substitutions, nucleotide deletions or insertions and double mutations (Supplementary Table S1). Several single nucleotide substitutions (at codons 103, 108, 119, 164 or 181) resulted in premature stop codons. Nucleotide deletions or insertions led to a shift in the reading frame and resulted in abnormal or early-truncated polypeptides. In addition, *pnc*A could not be amplified in five isolates (the experiment was repeated five times), suggesting that the gene was deleted, although this needs to be verified either by whole genome sequencing or amplification of a larger sequence covering the whole *pnc*A gene.^34, 35^ Finally, 30.3% (30/91) of mutants carried *pnc*A mutations in the three regions described as mutation hot spots (i.e., 3–17, 61–85 and 132–142)^14, 19^ (Supplementary Table S1).

With regards to the *pnc*A promoter mutations, the most common was a nucleotide substitution at position −11 (seven isolates). Nucleotide substitutions at positions −12, −13 and a 12-nucleotide deletion (from −18 to −7) were also detected (one isolate/each) (Supplementary Table S1).

### Association of FLD resistance patterns with *pnc*A mutations

Among the 205 FLD resistant isolates, 96 (46.8%) had at least one mutation in the *pnc*A gene or promoter, while 3 (5.5%) of the 55 FLD sensitive isolates had one mutation in the coding region (Table 1). This difference was statistically significant (*P*<5 × 10^−10^). Similarly, the *pnc*A mutation frequency in isolates resistant to a specific FLD (isoniazid, rifampicin, streptomycin and ethambutol) was significantly higher than in drug-sensitive isolates based on the odds ratio values (*P*<0.0001) (Table 2). Moreover, when comparing each drug resistance group, a Fisher's exact probability test results revealed significantly different mutation frequencies (*P*<0.0004). More specifically, the frequencies of mutations (Table 2) were significantly different between rifampicin, isoniazid and streptomycin drug-resistant isolates (*P*<0.015), and between ethambutol and isoniazid drug-resistant isolates (*P*<0.005).

When taking into account the FLD resistance patterns (mono to quadruple resistance), *pnc*A mutation frequency was the highest in quadruple-resistant isolates (75.8%), followed by triple-resistant (60.7%), double-resistant (32.0%), sensitive (5.5%) and mono-resistant isolates (3.3%) (Table 3). These differences were significant (*P*<10^−7^). The odds ratio calculation showed that, except in mono-drug resistant samples, the *pnc*A mutation frequency progressively increased from double- to quadruple-resistant isolates compared with FLD sensitive isolates (Table 3). Nevertheless, the differences were significant only between the mono-drug resistant patterns and all the other patterns (*P*<6 × 10^−4^) and between double- and quadruple-resistant patterns (*P*<8.4 × 10^−5^). In addition, the *pnc*A mutation frequency in MDR isolates was significantly higher than in non-MDR isolates (72.4% vs 7.2% *P*<10^−7^) (Table 3).

### Association of *pnc*A mutations with *M. tuberculosis* families and genotypes

The *pnc*A mutation frequency was highest in Beijing isolates (62 out of 130, 47.7%), followed by the T (9 out of 28, 32.1%), EAI (23 out of 76, 30.3%) and ‘Others’ isolates (5 out of 26, 19.2%). The global Fisher’s exact test was significant, supporting a difference between the Beijing and non-Beijing families (*P*<0.002). More specifically, the difference was significant between the Beijing and EAI families (*P*<0.018), and between the Beijing and ‘Others’ spoligotypes (*P*<0.008). Within the Beijing family, *pnc*A mutations were found in isolates showing all possible FLD resistance patterns. Among the MDR isolates, the *pnc*A mutation frequency was the highest in the T family (8/9, 88.9%), followed by Beijing (56 out of 76, 73.7%), EAI (21 out of 30, 70.0%) and ‘Others’ (4 out of 8, 50%). Nevertheless, the overall Fisher’s exact test revealed no significant differences (*P*>0.5). Because of the low sample size for the T, EAI family and ‘Others’ class compared with the Beijing isolates, the tests should be done on a larger sample.

Among the drug-resistant isolates, the frequency of *pnc*A mutations in the Beijing family (51.7%) was higher than in non-Beijing families (40.4%) but this difference was not significant (*P*=0.12). Among MDR isolates, the frequency of *pnc*A mutations in MDR Beijing isolates was slightly higher than in MDR non-Beijing families (73.7% and 70.2%, respectively) but was not significant. Similarly, among the drug-sensitive isolates, the frequency of *pnc*A mutations in the Beijing family was not significantly higher than in non-Beijing families (*P*=0.15).

The 99 *pnc*A mutants belonged to 79 different MIRU-VNTR/spoligotype genotypes, including 18 clusters. Most of these clusters included isolates showing various FLD resistance patterns and *pnc*A mutations (Supplementary Figure S1). For instance, in the largest MIRU-VNTR/spoligotype cluster (14 isolates), isolates harbored seven distinct *pnc*A mutations and six different FLD patterns. Finally, only two *pnc*A mutants had fully similar genetic and phenotypic patterns.

## DISCUSSION

### High diversity and frequency of *pnc*A mutations in clinical *M. tuberculosis* isolates in Vietnam

Our molecular analysis indicates that 38.1% of the clinical *M. tuberculosis* isolates selected in this study carry mutations in the *pnc*A gene or its promoter. A study in Northern Vietnam reported that 2.4% of isolates tested negative for PZase by a PZase assay and were considered to be resistant to PZA.^36^ However, as PZA-resistant isolates are not always PZase negative, it is thus difficult to compare the two studies.

In our study, *pnc*A mutations were very diverse and distributed throughout the gene without any one mutation predominating (71 different mutations detected in 50 different codons). This is in agreement with the fact that >600 distinct *pnc*A mutations in 171 out of 187 different codons have been identified so far.^18^ In our study, 30.3% of *pnc*A mutants carried mutations in the three previously described mutation hot spot regions.^14, 19^ Nevertheless, a recent meta-analysis showed that only 7.0% of PZA-resistant strains carry mutations in these regions.^18^ This underscores the great diversity of mutations at different positions that can lead to PZA resistance. Remarkably, for many *pnc*A mutations (85.0%), their link with PZA resistance has been experimentally confirmed (high confident mutations) and has been associated with high minimum inhibitory concentrations of PZA corresponding to high-level PZA resistance.^12, 17, 18, 19, 23, 24^ Furthermore, Stoffels *et al.* predicted by 3D structural analysis that most of the mutations detected in their PZA-resistant isolates affect PZA protein activity.^12^

Our data indicate that the diversity of mutations in the *pnc*A promoter is small (four mutations, 10 mutants), in agreement with the published data (34 mutations globally).^12, 17, 18, 32, 33^ These mutations can be associated with low- or high-level PZA resistance.

Finally, 55 of the 71 *pnc*A mutations detected in our samples were already known, with 50 having been previously linked to PZA resistance, and five found in either PZA-resistant/sensitive isolates or only in PZA-sensitive ones. Among the 16 novel mutations, 14 are likely to be linked to PZA resistance because of the mutation types or codon positions.^12, 17, 18, 32, 33^ For instance, a Met-Arg mutation was observed in codon 1 in our study while other changes, such as Met-Thr/Ile, were previously reported in this codon.^12, 37^ Similarly, new mutations were observed at codons 2 (Arg-Trp), 47 (Thr-Ile), 58 (Phe-Val), 82 (His-Gln), 142 (Thr-Arg) and 164 (Ser-Stop) in this study, while other mutations were previously described at the same codons, such as codons 2 (Arg-Arg), 47 (Thr-Ala/Pro/Ser), 58 (Phe-Ser/Leu), 82 (His-Tyr/Arg/Leu), 142 (Thr-Ala/Pro/Lys) and 164 (Ser-Pro).^19, 38, 39, 40, 41, 42^

Altogether, 90.1% (64/71) of the *pnc*A mutations identified in our study are likely to be associated with PZA resistance. Thus, out of 99 mutants, 91 (92%) carried high-confidence *pnc*A mutations linked to PZA resistance, 2 carried mutations with an unknown link to PZA resistance and 6 are probably not linked to PZA resistance.

### *Pnc*A mutation frequency and FLD resistance patterns

In our study, three FLD sensitive isolates carried *pnc*A mutations located in the PZase active or metal-binding site. These isolates could be mono-PZA resistant, especially the two carrying mutations at codons 49 and 138, which have been linked to high-level PZA resistance.^12, 13, 19^ In agreement with this observation, a recent study reported a significant proportion of phenotypic mono-PZA resistance (4.2%) in clinical isolates.^23^ Nevertheless, as expected, the *pnc*A mutation frequency was significantly higher in FLD-resistant isolates compared to FLD-sensitive ones.

A recent study showed an association between *pnc*A mutation frequency and rifampicin resistance, although a link between *pnc*A mutations and resistance to other FLD has not yet been reported.^11^ In our study, we found that the frequency of *pnc*A mutations was significantly different between each drug resistance group (isoniazid, rifampicin, streptomycin and ethambutol resistance) and the corresponding sensitive isolates, suggesting an association between PZA resistance and each FLD drug resistance.

Analyses according to FLD resistance patterns suggest that the frequency of *pnc*A gene mutations progressively increases with the FLD resistance (mono, double, triple and quadruple resistance). Quadruple-resistant isolates showed the highest *pnc*A mutation rate (75.8%). Similarly, the *pnc*A mutation frequency was also very high in MDR isolates (72.4%), consistent with many other studies performed worldwide,^12, 23, 24, 43^ suggesting a cumulative effect of drug resistance mutations. Another study showed that the proportion of PZA-resistant strains is higher in pre-XDR and XDR than in MDR isolates.^44^ Altogether, these data suggest a higher chance of detecting PZA resistance in more severely drug-resistant *M. tuberculosis* isolates. This is very worrying because PZA resistance in MDR and XDR TB is generally associated with poor treatment outcomes.^6, 7^ Thus, PZA susceptibility testing prior to treatment is a priority, especially in patients with MDR and XDR TB.

### *Pnc*A mutation frequency is highest in the Beijing family

The genotyping analysis did not highlight any association between specific *pnc*A mutations and the different *M. tuberculosis* families or MIRU-VNTR clusters, in agreement with other studies.^12, 43, 45^ Nevertheless, the mutation frequency was higher in Beijing isolates compared with other families or genotypes, suggesting a greater association of this family with PZA resistance. Furthermore, *pnc*A mutations in this family were found in FLD susceptible to quadruple-resistant isolates. In the other families, mutations were particularly detected in triple- and quadruple-resistant isolates. We thus hypothesize that the Beijing family may be more susceptible to acquiring *pnc*A mutations, whatever the resistance profile, which should be experimentally demonstrated. Although the situation differs by country,^23, 43, 45^ the Beijing family seems to be strongly linked to PZA resistance in Vietnamese samples. This is all the more worrying since the Beijing family is currently spreading in Vietnam.^46^

### Evolution of PZA resistance in *M. tuberculosis* population

The observed *pnc*A mutation diversity and frequency was very high in our study. This can be attributed to several factors, including a high rate of PZA resistance acquisition (10^−5^ bacilli *in vitro*),^12^ the non-necessity of *pnc*A (*M. tuberculosis* can survive and grow without this gene)^35^ and the absence of a loss of bacterial fitness in the presence of *pnc*A mutations.^12^ This peculiar evolution of the *pnc*A gene may lead to the emergence of PZA resistance at a high frequency, thus limiting the use of PZA as a key drug in current and future treatment regimens, despite its powerful sterilizing abilities. Because of a general lack of clustering of *pnc*A mutants in clinical isolates, some researchers have hypothesized that *pnc*A mutations bear high fitness costs, impairing the transmission of *M. tuberculosis*.^47^ However, the reported spread of some MDR and XDR clones carrying specific *pnc*A mutations refutes this hypothesis.^48, 49^ The study of PZA resistance mechanisms and tracking its evolution are crucial for TB control.

## Figures and Tables

**Table 1:** Distribution of first-line drug susceptibility patterns according to the *Mycobacterium tuberculosis* families and the relative frequency of *pnc*A mutations

**Type**	**Drug susceptibility patterns**	***M. tuberculosis*** **families**				**Total**	***pnc*****A mutation *n* (%)**
		**EAI**	**Beijing**	**T**	**‘Others’**		
FLD sensitive	Sensitive	20	14	11	10	55	3 (5.5)
Mono-resistant	H	12	11	2	2	27	2 (7.4)
	R	0	0	0	2	2	0 (0)
	S	11	14	4	2	31	0 (0)
	E	0	1	0	0	1	0 (0)
Double-resistant	HS	3	12	2	2	19	5 (26.3)
	HR	4	1	0	1	6	3 (50)
Triple-resistant	HRS	5	11	1	3	20	12 (60)
	HRE	4	2	0	0	6	5 (83.3)
	HSE	0	1	0	0	1	0 (0)
	RSE	0	1	0	0	1	0 (0)
Quadruple-resistant	HRSE	17	62	8	4	91	69 (75.8)
Total		76	130	28	26	260	99 (38.1)

Abbreviations: resistant to isoniazid, H; resistant to rifampicin, R; resistant to streptomycin, S; resistant to ethambutol, E; sensitive to all four first-line drugs (FLDs) (isoniazid, rifampicin, streptomycin and ethambutol), FLD sensitive.

‘Others’: Include 1 H, 3 LAM and 22 unknown isolates.

Mono-resistant: isolates resistant to H or R, S, E; double-resistant: isolates resistant to two of the four FLDs; triple-resistant: isolates resistant to three FLDs; and quadruple-resistant: isolates resistant to all four FLDs.

**Table 2:** Comparison of the *pnc*A mutation frequencies of isoniazid, rifampin, streptomycin or ethambutol resistant isolates and of the corresponding sensitive isolates

**Resistant/sensitive pattern**	**Number** **of isolates**		**Related frequency of** ***pnc*****A mutation (%)**	**Odds ratio, 95% CI**	***P*****-value**
	**With** ***pnc*****A mutation**	**Without** ***pnc*****A mutation**			
Isoniazid-resistant	96	74	56.5	37.6, 11.4–123.7	*P*<0.0001
Isoniazid-sensitive	3	87	3.3		
Rifampicin-resistant	89	37	70.6	29.8, 14.1–63.1	*P*<0.0001
Rifampicin-sensitive	10	124	7.5		
Streptomycin-resistant	86	77	52.8	7.2, 3.7–14.0	*P*<0.0001
Streptomycin-sensitive	13	84	13.4		
Ethambutol-resistant	74	26	74	15.4, 8.3–28.5	*P*<0.0001
Ethambutol-sensitive	25	135	15.6		

**Table 3:** Comparison of the *pnc*A mutation frequencies of isolates with different drug-resistant patterns (sensitive, mono-, double-, triple- and quadruple-resistant) and first-line drug sensitive isolates

**Type of isolates**	**Number** **of isolates**		**Related frequency of** ***pnc*****A mutation (%)**	**Odds ratio, 95% CI**	***P*****-value**
	**With mutation**	**Without mutation**			
Sensitive	3	52	5.5	—	—
Mono-resistant	2	59	3.3	0.6, 0.1–3.7	*P*>0.5
Double-resistant	8	17	32	8.2, 1.9–34.3	*P*<0.005
Triple-resistant	17	11	60.7	26.8, 6.7–107.5	*P*<0.001
Quadruple-resistant	69	22	75.8	54.4, 15.4–191.4	*P*<0.001
MDR	89	34	72.4	45.4, 13.3–155.1	*P*<0.0001
Non MDR	10	127	7.9	1.4, 0.4–5.2	*P*>0.5
