# Supplementary material for: Molecular analysis of pyrazinamide resistance in Mycobacterium tuberculosis in Vietnam highlights the high rate of pyrazinamide resistance-associated mutations in clinical isolates
Source: Emerg Microbes Infect. 2017 Oct 11;6(10):e86–. doi: 10.1038/emi.2017.73 (PMC5658769; doi:10.1038/emi.2017.73)
Supplement: Supplementary Table S1 [file emi201773x2.docx]

**Supplementary Table S1** Mutations found in *pnc*A gene and its promoter of the studied Vietnamese *Mycobacterium tuberculosis* isolates. The table shows the diversity of *pnc*A mutations (71 different types), the mutation positions and their nucleotide/amino acid change(s) and characteristics of mutations (reported/unreported in the literature). The table also presents the mutations found in PZA-resistant and/or PZA-susceptible isolates and its association with PZA resistance/susceptibility based on literature review.

| **No** | **DST pattern** | **No of isolate(s)** | **Nucleotide position(s)** | **Codon position(s)** | **Nucleotide substitution(s)** | **Amino acid change(s)** | **Mutations reported/unreported in the literature** | **PZA Resistant/sensitive isolates** | **Linked to PZA resistant/sensitive** | **Reference** |
| --- | --- | --- | --- | --- | --- | --- | --- | --- | --- | --- |
| 1 | HS | 7 | -11 | NA | A-G | NA | Yes | R & S | R | Mittio et al. 2014 & Ramirez-Busby et al. 2015 |
| 2 | HRSE | 1 | -12 | NA | T-G | NA | Yes | R | R | Whitfield et al.2015 |
| 3 | H | 1 | -13 | NA | G-T | NA | Yes | R & S | R | Mittio et al. 2014 & Ramirez-Busby et al. 2015 |
| 4 | HRS | 1 | (-18)-(-7) | NA | Deletion CGAACGTATGGT | NA | Novel deletion | U | R | New |
| 5 | HRSE | 1 | 2 | 1 | ATG-AGG | Met-Arg (frameshift) | New amino acid replacement | U | R | New |
| 6 | HRS | 1 | 4 | 2 | CGG-TGG | Arg-Trp | New amino acid replacement | U | U | New |
| 7 | HRE | 1 | 16 | 6 | ATC-CTC | Ile-Leu | Yes | R & S | S | Mittio et al. 2014 & Ramirez-Busby et al. 2015 |
| 8 | HRS | 1 | 17 | 6 | ATC-ACC | Ile-Thr | Yes | R | R | Mittio et al. 2014 & Ramirez-Busby et al. 2015 |
| 9 | HRS | 1 | 19 | 7 | GTC-TTC | Val-Phe | Yes | R | R | Mittio et al. 2014 & Ramirez-Busby et al. 2015 |
| 10 | HRS | 1 | 29 | 10 | CAG-CCG | Gln-Pro | Yes | R | R | Mittio et al. 2014 & Ramirez-Busby et al. 2015 |
| 11 | HRSE | 1 | 29 | 10 | CAG-CGG | Gln-Arg | Yes | R & S | R | Mittio et al. 2014 & Ramirez-Busby et al. 2015 |
| 12 | HR | 1 | 40 | 14 | TGC-CGC | Cys-Arg | Yes | R | R | Mittio et al. 2014 & Ramirez-Busby et al. 2015 |
| 13 | HRSE | 2 | 55 | 19 | del C | frameshift | Novel deletion | U | R | New |
| 14 | HRSE | 1 | 104 | 35 | CTG-CCG | Leu-Pro | Yes | R | R | Mittio et al. 2014 |
| 15 | HRSE | 1 | 139 | 47 | ACC-GCC | Thr-Ala | Yes | R & S | S | Mittio et al. 2014 |
| 16 | HRE | 1 | 140 | 47 | ACC-ATC | Thr-Ile | New amino acid replacement | U | U | New |
| 17 | HRSE | 1 | 146 | 49 | GAC-GCC | Asp-Ala | Yes | R | R | Mittio et al. 2014 |
| 18 | Sensitive, HRSE | 2 | 147 | 49 | GAC-GAG | Asp-Glu | Yes | S | S | Bhuju et al. 2013 |
| 19 | HRSE | 1 | 151 | 51 | CAC-TAC | His-Tyr | Yes | R | R | Mittio et al. 2014 |
| 20 | HRSE | 1 | 152 | 51 | CAC-CGC | His-Arg | Yes | R | R | Mittio et al. 2014 |
| 21 | HRSE | 1 | 161 | 54 | CCG-CAG | Pro-Gln | Yes | R | R | Mittio et al. 2014 |
| 22 | HRSE | 1 | 161 | 54 | CCG-CTG | Pro-Leu | Yes | R | R | Mittio et al. 2014 |
| 23 | HRSE | 1 | 169 | 57 | CAC-TAC | His-Tyr | Yes | R | R | Mittio et al. 2014 |
| 24 | HRSE | 1 | 170 | 57 | CAC-CCC | His-Pro | Yes | R | R | Mittio et al. 2014 |
| 25 | HRSE | 1 | 172 | 58 | TTC-GTC | Phe-Val | New amino acid replacement | U | R | New |
| 26 | HRSE | 2 | 173 | 58 | TTC-TCC | Phe-Ser | Yes | R | R | Ramirez-Busby et al. 2015 |
| 27 | HRSE | 1 | 176 | 59 | TCC-TTC | Ser-Phe | Yes | S | S | Campbel et al. 2011, Xia et al. 2015 |
| 28 | HRS | 1 | 181 | 61 | ACA-CCA | Thr-Pro | Yes | R | R | Ramirez-Busby et al. 2015 |
| 29 | HRSE | 2 | 184 | 62 | CCG-TCG | Pro-Ser | Yes | R | R | Ali et al. 2015 |
| 30 | HRSE | 1 | 185 | 62 | CCG-CTG | Pro-Leu | Yes | R | R | Mittio et al. 2014 |
| 31 | HR | 1 | 188 | 63 | GAC-GGC | Asp-Gly | Yes | R | R | Mittio et al. 2014 |
| 32 | HRSE | 1 | 188 | 63 | GAC-GCC | Asp-Ala | Yes | R | R | Mittio et al. 2014 & Ramirez-Busby et al. 2015 |
| 33 | HRSE | 2 | 202 | 68 | TGG-CGG | Trp-Arg | Yes | R | R | Mittio et al. 2014 & Ramirez-Busby et al. 2015 |
| 34 | HRE | 2 | 226 | 76 | ACT-CCT | Thr-Pro | Yes | R | R | Mittio et al. 2014 & Ramirez-Busby et al. 2015 |
| 35 | HRSE | 1 | 246 | 82 | CAT-CAA | His-Gln | New amino acid replacement | U | R | New |
| 36 | HRSE | 3 | 286 | 96 | AAG-GAG | Lys-Glu | Yes | R | R | Mittio et al. 2014 & Ramirez-Busby et al. 2015 |
| 37 | HRS | 1 | 287 | 96 | AAG-ACG | Lys-Thr | Yes | R | R | Mittio et al. 2014 & Ramirez-Busby et al. 2015 |
| 38 | HRSE | 1 | 305 | 102 | GCG-GTG | Ala-Val | Yes | R & S | S | Mittio et al. 2014 & Ramirez-Busby et al. 2015 |
| 39 | HR | 2 | 308 | 103 | TAC-TCC | Tyr-Ser | Yes | R | R | Ramirez-Busby et al. 2015 |
| 40 | HRSE | 1 | 309 | 103 | TAC-TAG | Tyr-Stop | Yes | R | R | Mittio et al. 2014 |
| 41 | HRSE | 2 | 312 | 104 | AGC-AGA | Ser-Arg | Yes | R | R | Mittio et al. 2014 & Ramirez-Busby et al. 2015 |
| 42 | HRSE | 1 | 322 | 108 | GGA-TGA | Gly-Stop | Yes | R | R | Ramirez-Busby et al. 2015 |
| 43 | HRSE | 1 | 347 | 116 | CTG-CCG | Leu-Pro | Yes | R | R | Mittio et al. 2014 & Ramirez-Busby et al. 2015 |
| 44 | HS | 1 | 357 | 119 | TGG-TGT | Trp-Cys | Yes | R | R | Mittio et al. 2014 |
| 45 | HRSE | 1 | 357 | 119 | TGG-TGA | Trp-Stop | Yes | R | R | Mittio et al. 2014 & Ramirez-Busby et al. 2015 |
| 46 | HRE | 1 | 359 | 120 | CTG-CCG | Leu-Pro | Yes | R | R | Mittio et al. 2014 & Ramirez-Busby et al. 2016 |
| 47 | HS | 1 | 383 | 128 | GTC-GGC | Val-Gly | Yes | R | R | Mittio et al. 2014 & Ramirez-Busby et al. 2017 |
| 48 | HRS | 2 | 389 | 130 | GTG-GGG | Val-Gly | Yes | R | R | Mittio et al. 2014 & Ramirez-Busby et al. 2018 |
| 49 | HRSE | 1 | 401 | 134 | GCC-GTC | Ala-Val | Yes | R | R | Mittio et al. 2014 & Ramirez-Busby et al. 2019 |
| 50 | HRSE | 1 | 410 | 137 | CAT-CGT | His-Arg | Yes | R | R | Mittio et al. 2014 & Ramirez-Busby et al. 2020 |
| 51 | HRSE | 1 | 412 | 138 | TGT-CGT | Cys-Arg | Yes | R | R | Mittio et al. 2014 & Ramirez-Busby et al. 2021 |
| 52 | Sensitive, HRSE | 2 | 412 | 138 | TGT-AGT | Cys-Ser | Yes | R | R | Ramirez-Busby et al. 2015 |
| 53 | HRSE | 2 | 422 | 141 | CAG-CCG | Gln-Pro | Yes | R | R | Mittio et al. 2014, Ramirez-Busby et al. 2015 |
| 54 | HS | 2 | 424 | 142 | ACG-GCG | Thr-Ala | Yes | R | R | Mittio et al. 2014, Ramirez-Busby et al. 2015 |
| 55 | HRSE | 1 | 425 | 142 | ACG-AGG | Thr-Arg | New amino acid replacement | U | R | New |
| 56 | HRSE | 1 | 436 | 146 | GCG-CCG | Ala-Pro | Yes | R | R | Ramirez-Busby et al. 2015 |
| 57 | H | 1 | 446 | 149 | AAT-AGT | Asn-Ser | New amino acid replacement | U | R | New |
| 58 | HRSE | 1 | 460 | 154 | AGG-GGG | Arg-Gly | Yes | R | R | Mittio et al. 2014, Ramirez-Busby et al. 2015 |
| 59 | HR | 3 | 464 | 155 | GTG-GGG | Val-Gly | Yes | R | R | Mittio et al. 2014, Ramirez-Busby et al. 2015 |
| 60 | HRSE | 1 | 467 | 156 | CTG-CCG | Leu-Pro | Yes | R | R | Mittio et al. 2014, Ramirez-Busby et al. 2015 |
| 61 | HRSE | 1 | 491 | 164 | TCG-TAG | Ser-Stop | New amino acid replacement | U | R | New |
| 62 | HRSE | 1 | 541 | 181 | GAG-TAG | Glu-Stop | New amino acid replacement | U | R | New |
| 63 | HRSE | 1 | 187-188 & 319 | 63 & 107 | Insertion ACTATTCCTC & GAA-AAA | frameshift and Glu-Lys | New insertion and new mutation pattern | U | R | New |
| 64 | HRSE | 1 | 231-232 | 78 | Insertion G | frameshift | Yes | R | R | Mittio et al. 2014, Ramirez-Busby et al. 2015 |
| 65 | HRSE | 1 | 290 & 548 | 97 & 183 | GGT-GAT & deletion T | Gly-Asp & frameshift | New deletion and new mutation pattern | U | R | New |
| 66 | HRSE | 2 | 389-390 | 130 | insertion GG | frameshift | Yes | R | R | Whitfield et al.2015 |
| 67 | HRSE | 2 | 482-483 | 161 | Insertion G | frameshift | New insertion | U | R | New |
| 68 | HRSE | 1 | 491-492 | 164 | Insertion C | frameshift | New insertion | U | R | New |
| 69 | HS | 1 | 82 &418-419 | 28 & 140 | GCC-ACC & deletion G | Ala-Thr & frameshift | New deletion and new mutation pattern | U | R | New |
| 70 | HRE | 5 | Whole pncA deletion | Whole pncA deletion | Whole pncA deletion | Whole pncA deletion | Yes | R | R | Aono et al. 2014, Martinez et al. 2015 |
| 71 | Susceptible | 1 | 307 | 103 | TAC-GAC | Tyr-Asp | Yes | R | R | Mittio et al. 2014, Ramirez-Busby et al. 2015 |

H: resistant to isoniazid; R: resistant to rifampin; S: resistant to streptomycin; E: resistant to ethambutol; NA: mutation in *pnc*A promoter region; ^#^: references [^1-5^](#_ENREF_1).

1. Stoffels K, Mathys V, Fauville-Dufaux M, Wintjens R, Bifani P. Systematic analysis of pyrazinamide-resistant spontaneous mutants and clinical isolates of *Mycobacterium tuberculosis*. *Antimicrob Agents Chemother* 2012 Oct; **56**(10)**:** 5186-5193.

2. Miotto P, Cabibbe AM, Feuerriegel S, Casali N, Drobniewski F, Rodionova Y*, et al.* *Mycobacterium tuberculosis* pyrazinamide resistance determinants: a multicenter study. *MBio* 2014; **5**(5)**:** e01819-01814.

3. Ramirez-Busby SM, Valafar F. Systematic review of mutations in pyrazinamidase associated with pyrazinamide resistance in *Mycobacterium tuberculosis* clinical isolates. *Antimicrob Agents Chemother* 2015 Sep; **59**(9)**:** 5267-5277.

4. Sandgren A, Strong M, Muthukrishnan P, Weiner BK, Church GM, Murray MB. Tuberculosis drug resistance mutation database. *PLoS Med* 2009 Feb 10; **6**(2)**:** e2.

5. Whitfield MG, Soeters HM, Warren RM, York T, Sampson SL, Streicher EM*, et al.* A Global Perspective on Pyrazinamide Resistance: Systematic Review and Meta-Analysis. *PLoS One* 2015; **10**(7)**:** e0133869.

6. Akio Aono, Kinuyo Chikamatsu, Hiroyuki Yamada,Tomoko Kato, Satoshi Mitarai. Association between *pnc*A Gene Mutations, Pyrazinamidase Activity, and Pyrazinamide Susceptibility Testing in *Mycobacterium tuberculosis*. Antimicrob Agent and Chemo 2014; 58(8): 4928–4930

7. Patricia J. Campbell, Glenn P. Morlock, R. David Sikes, Tracy L. Dalton, Beverly Metchock, Angela M. Starks, Delaina P. Hooks, Lauren S. Cowan, Bonnie B. Plikaytis, and James E. Posey. Molecular Detection of Mutations Associated with First- and Second-Line Drug Resistance Compared with Conventional Drug Susceptibility Testing of *Mycobacterium tuberculosis*. Antimicrob Agent and Chemo 2011; 55 (5): 2032–2041.

8. Qiang Xia, Li-li Zhao, Feng Li, Yu-mei Fan, Yuan-yuan Chen, Bei-bei Wu,Zheng-wei Liu, Ai-zhen Pan, Min Zhu. Phenotypic and Genotypic Characterization of Pyrazinamide Resistance among Multidrug-Resistant Mycobacterium tuberculosis Isolates in Zhejiang, China. Antimicrob Agent and Chemo 2011; 59 (3): 1690–1695.
