# Supplementary material for: Molecular analysis of pyrazinamide resistance in Mycobacterium tuberculosis in Vietnam highlights the high rate of pyrazinamide resistance-associated mutations in clinical isolates
Source: Emerg Microbes Infect. 2017 Oct 11;6(10):e86–. doi: 10.1038/emi.2017.73 (PMC5658769; doi:10.1038/emi.2017.73)
Supplement: Supplementary Figure S1 [file emi201773x1.pdf]

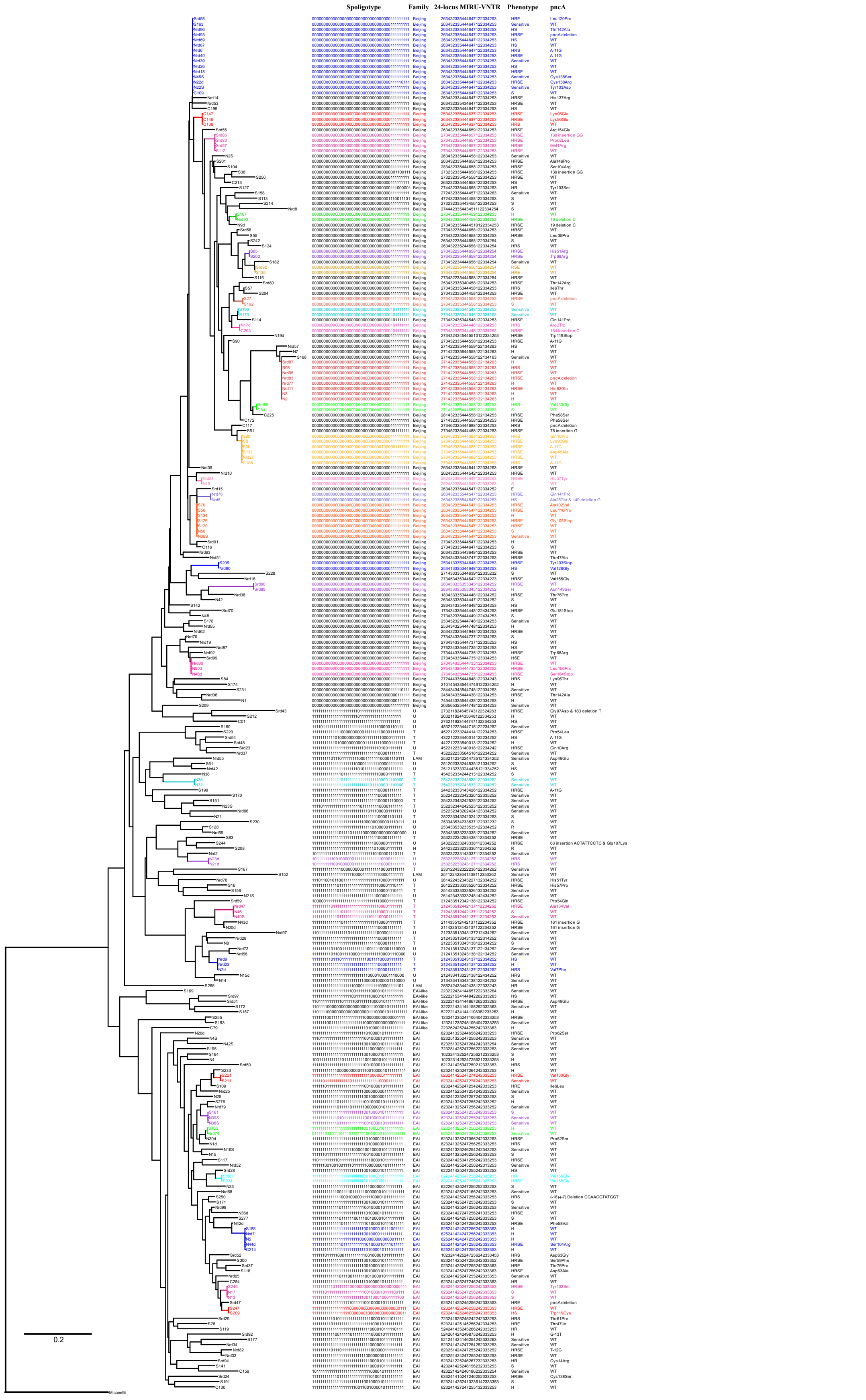

Supplementary Table S1 Phylogenetic tree based on 24-locus MIRU-VNTR data of the 260 *Mycobacterium tuberculosis* isolates in Vietnam
